# Supramolecular Cationic Assemblies against Multidrug-Resistant Microorganisms: Activity and Mechanism of Action

**DOI:** 10.3390/ijms16036337

**Published:** 2015-03-19

**Authors:** Letícia Dias de Melo Carrasco, Jorge Luiz Mello Sampaio, Ana Maria Carmona-Ribeiro

**Affiliations:** 1 Departamento de Bioquímica, Instituto de Química, Universidade de São Paulo, Caixa Postal 26077, CEP 05513-970 São Paulo, Brazil; E-Mail: lemelodias@usp.br; 2 Departamento de Análises Clínicas e Toxicológicas, Faculdade de Ciências Farmacêuticas, Universidade de São Paulo, CEP 05508-900 São Paulo, Brazil; E-Mail: sampaio@usp.br

**Keywords:** multidrug-resistant microbes, viable cells counting, broad-spectrum and antimicrobial activity, biocompatible cationic polymer, self-assembled, cationic and hybrid nanoparticles, nanoparticles disassembly, leakage of intracellular compounds, damage to the cell wall, scanning electron microscopy

## Abstract

The growing challenge of antimicrobial resistance to antibiotics requires novel synthetic drugs or new formulations for old drugs. Here, cationic nanostructured particles (NPs) self-assembled from cationic bilayer fragments and polyelectrolytes are tested against four multidrug-resistant (MDR) strains of clinical importance. The non-hemolytic poly(diallyldimethylammonium) chloride (PDDA) polymer as the outer NP layer shows a remarkable activity against these organisms. The mechanism of cell death involves bacterial membrane lysis as determined from the leakage of inner phosphorylated compounds and possibly disassembly of the NP with the appearance of multilayered fibers made of the NP components and the biopolymers withdrawn from the cell wall. The NPs display broad-spectrum activity against MDR microorganisms, including Gram-negative and Gram-positive bacteria and yeast.

## 1. Introduction

Nanoparticles have some unique properties for drug delivery and human healthcare. Their nanoscale size and large surface area/mass ratios can reduce drug dose and toxicity, improving the drug therapeutic index [1,2,3,4]. Bioactive and biocompatible nanoparticles have been obtained either by synthetic procedures for covalent modification of biocompatible molecules [2,3] or by the self-assembly of diverse materials [3]. Polymeric nanoparticles are among the most studied carriers for drugs with potential for a variety of medical applications, including antimicrobial therapy [5]. In particular, the microbial resistance to antibiotics by mutation, adaptation or acquisition of plasmids or other genetic elements [6,7] with the occurrence of thicker cell walls, reduced antibiotic uptake or active antibiotic removal by efflux pumps [8,9], culminates in the appearance of multidrug-resistant (MDR) strains requiring novel drugs or new formulations for old drugs [6,10].

Bacteria and fungi are negatively-charged and interact with a variety of positively-charged compounds, such as surfactants, lipids, polymers or peptides, which eventually act as antimicrobial agents [10,11,12]. The permanently-charged quaternary ammonium moiety is frequently used to impart the antimicrobial property to many surfactants [13] and polymers [5]. A variety of quaternary ammonium compounds (QAC) has been covalently bound or embedded in nanoparticles [14,15,16,17]. QAC are perfectly suitable for disinfection; however, their toxicity may hamper their use *in vivo*. Several nanomaterials based on polyelectrolytes and their nanocomposites have been developed for antibacterial applications [18,19,20]. The cationic polymer, poly (diallyldimethylammonium) chloride (PDDA), with pendant quaternary nitrogen groups, does not cause hemolysis [16] and can easily be formulated as the outer layer of self-assembled nanoparticles (NPs) [16,17,21,22]. PDDA applications range from the paper, mining and food to water treatment industries, displaying a very potent bactericidal and fungicidal activity [16,17]. We have previously characterized some self-assembled NPs based on cationic bilayer fragments (BFs) of dioctadecyldimethylammonium bromide (DODAB) [23,24] surrounded by a layer of carboxymethylcellulose (CMC) and a second layer of the PDDA antimicrobial polymer [16,22]. These self-assembled NPs display high antimicrobial activity against bacteria and fungi at low PDDA concentrations [16,17,21,22], but were not tested before against MDR strains. Here, the DODAB BF/CMC/PDDA NPs are evaluated against four clinical isolates of MDR pathogens showing outstanding microbicidal activity. In addition, the mechanism of action for the NPs against the cells is clarified by determining the leakage of intracellular phosphorylated compounds from the microbial cells and evaluating the effects of NPs on the cells by scanning electron microscopy (SEM). The leakage of phosphorylated compounds suggested cell membrane lysis. The SEM micrographs suggested the disassembly of the self-assembled NPs and the appearance of novel assemblies in the form of fibers and/or multilayered bundles protruding from the cells, which possibly included the NP polymers and the biopolymers withdrawn from the cell wall.

## 2. Results and Discussion

The construction of the nanostructured particles (NPs) of DODAB BF/CMC/PDDA employed the layer-by-layer technique [25]. The formation of NPs by self-assembly was driven by the electrostatic attraction between positively-charged bilayer fragments and anionic CMC plus the electrostatic attraction between CMC and PDDA. The cationic DODAB BFs were sequentially covered by the anionic polyelectrolyte, CMC, and the cationic antimicrobial polymer, PDDA [16,17,21,22]. Table 1 shows the zeta-average diameter (Dz) or mean hydrodynamic diameter, polydispersity (P) and zeta-potential (ζ) of the assemblies used in the construction of NPs. Dz, ζ and P for the NPs are 108 ± 1 nm, 37 ± 2 mV and 0.131 ± 0.010, respectively, and consistent with the previous data for the same NPs [16].

**Table 1:** Zeta-average diameter (Dz), zeta-potential (ζ) and polydispersity (P) of dioctadecyldimethylammonium bromide (DODAB) bilayer fragments (BFs), DODAB BF/carboxymethylcellulose (CMC) and DODAB BF/CMC/poly(diallyldimethylammonium) chloride (PDDA) assemblies or NPs.

Dispersion	[DODAB] (mM)	[CMC] (mg/mL)	[PDDA] (mg/mL)	Dz (nm)	ζ (mV)	P
DODAB BF	1.0	-	-	81 ± 1	52 ± 3	0.172 ± 0.010
DODAB BF/CMC	0.1	0.1	-	114 ± 1	−43 ± 2	0.125 ± 0.010
DODAB BF/CMC/PDDA	0.1	0.1	0.1	108 ± 1	37 ± 2	0.131 ± 0.010

**Figure 1:**
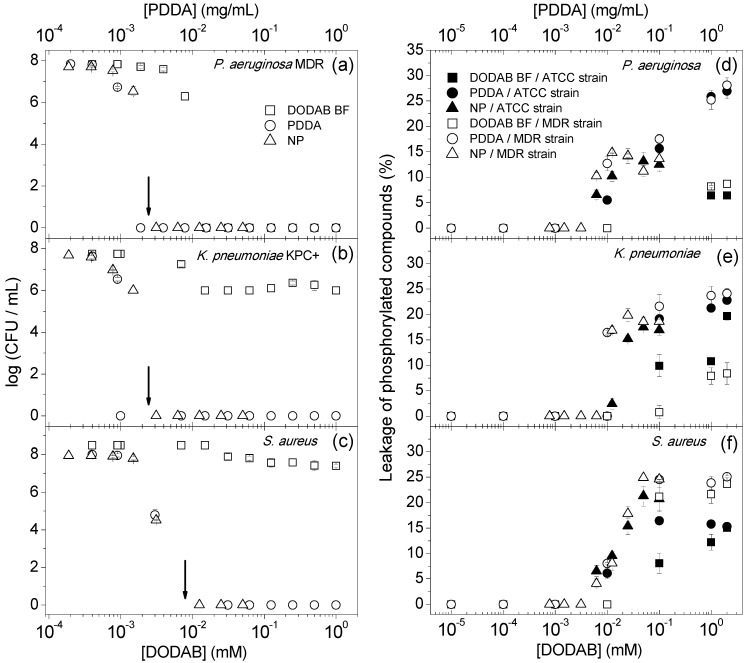
(**a**–**c**) Cell viability as a function of DODAB or PDDA concentrations for the *P. aeruginosa* multidrug-resistant (MDR) strain (5–7 × 10^7^ CFU/mL), *K. pneumoniae* KPC+ (*K. pneumoniae* carbapenemase (KPC+)) (4–5 × 10^7^ CFU/mL) or MRSA (8 × 10^7^–3 × 10^8^ CFU/mL); (**d**–**f**) leakage of phosphorylated compounds (%) as a function of DODAB or PDDA concentrations for reference (ATCC) or MDR *P. aeruginosa* (2 × 10^8^–5 × 10^10^ CFU/mL), *K. pneumoniae* (3 × 10^8^–1 × 10^10^ CFU/mL) or *S. aureus* (5 × 10^9^–6 × 10^11^ CFU/mL). Interactions took place for 1 h. The minimal microbicidal concentration (MMC) values for PDDA in NPs are indicated by arrows.

Figure 1 shows the antibacterial activity of the NPs against the MDR bacterial strains in comparison with the controls for DODAB BF or PDDA alone. From the sigmoidal dependencies of the log (colony forming unities (CFU)/mL) on DODAB or PDDA concentrations, there is an eight-log reduction caused by NPs or PDDA at and above the minimal microbicidal concentration (MMC) (Figure 1a–c). Against *Pseudomonas aeruginosa* MDR, the DODAB BF effect is similar to the one of NPs or PDDA (Figure 1a). Against *Klebsiella pneumoniae*, the producer of *K. pneumoniae* carbapenemase (KPC+), and methicillin-resistant *Staphylococcus aureus* (MRSA), DODAB BF causes a two- and one-log reduction in cell viability, respectively (Figure 1b,c), while PDDA and NP cause an eight-log reduction in cell viability at and above the MMC. In Figure 1d–f, the leakage of intracellular phosphorylated compounds induced by the cationic assemblies at 10^9^ bacteria/mL as a function of DODAB or PDDA concentrations elucidates the mechanism of action for NPs. Over a range of low DODAB or PDDA concentrations, no leakage is observed. Above 6 × 10^−3^ mg/mL PDDA, the leakage increases with PDDA concentrations for all bacterial strains tested. One should notice that despite the nanomolar sensitivity of the method used to determine the leakage of phosphorylated compounds from the microbial cells [26], it was not possible to detect the leakage from 10^7^ CFU/mL of bacterial cells, the appropriate cell concentration for performing the MMC determination. Therefore, cell concentrations about 10–100-times higher than those in Figure 1a–c were used to obtain the results in Figure 1d–f.

**Figure 2:**
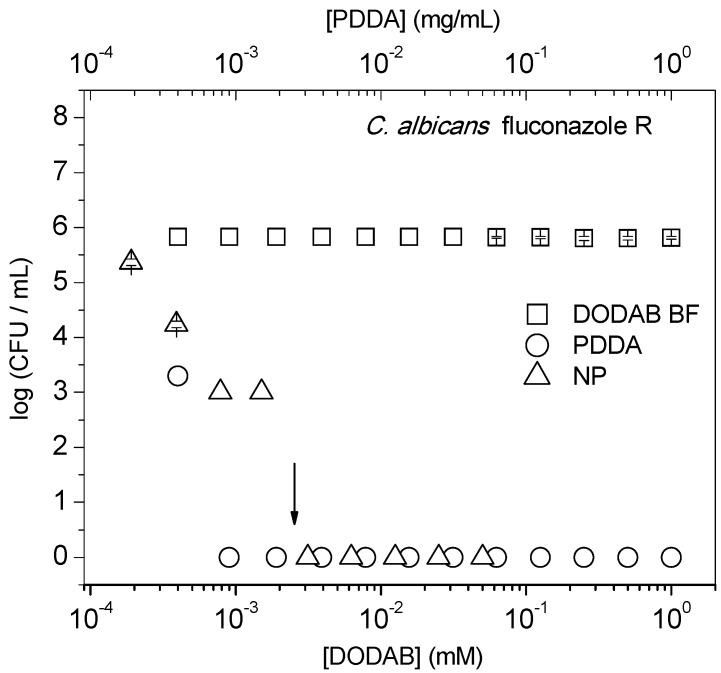
The cell viability of fluconazole-resistant *C. albicans* at 6–8 × 10^5^ CFU/mL as a function of DODAB or PDDA concentrations. Interactions took place for 1 h. The MMC for PDDA in the NPs is indicated by the arrow.

Figure 2 shows the important fungicidal activity of the NPs and PDDA against a clinical isolate of fluconazole-resistant (R) *Candida albicans*. There is a six-log reduction of cell viability from the MMC, which contrasts with the absence of activity observed for DODAB BF only.

MMC values for DODAB alone, PDDA alone or PDDA in NPs against the MDR bacteria and yeast are in Table 2. DODAB is a bactericidal agent against the *P. aeruginosa* MDR strain from 10 μM. PDDA by itself showed a potent microbicidal action against all strains tested at very small concentrations (Table 2). The most sensitive strain is the resistant fungus, which requires only 0.8 μg/mL of PDDA alone to be killed. PDDA in NPs is effective at slightly larger doses (2.5 μg/mL PDDA) than the one for PDDA alone. Similar results are observed also for the bacteria. The reason for this is partial neutralization of the cationic charges at the outer layer by the underlying anionic CMC layer in the NP structure, so that not all PDDA cationic charges in the NPs are available to interact with the cells. Curiously, the Gram-positive MRSA requires the highest PDDA concentration to be killed, 5 and 8 μg/mL PDDA alone or in the NPs, respectively. NPs are more efficient against Gram-negative bacteria and yeast than against Gram-positive bacteria.

The NPs antimicrobial activity against the MDR microorganisms is more appropriately reported as the log CFU/mL (Figure 1 and Figure 2) in contrast to other less effective ways of presenting cell viability, such as the percentile of viable cells (% CFU/mL) [16,17,21,22,27,28,29,30,31,32]. For example, starting at cell concentrations of around 10^8^ and 10^5^ CFU/mL for bacteria and yeast, respectively, 99.9% of cell death represents a three-log reduction in cell viability with about 10^5^ and 10^2^ cells/mL still viable, respectively. The percentile of viable cells does not allow discriminating between a potent agent yielding an eight-log reduction and a moderate agent yielding only a three-log reduction. A three-log reduction on viability has been accepted as an effective antimicrobial or even microbicidal intervention for disinfectants [33,34]. Therefore, that PDDA or NPs induced a reduction of eight- and five-log on the cell viability is remarkable (Figure 1 and Figure 2). On the other hand, DODAB BF dispersions yield a two- or one-log reduction against *K. pneumoniae* KPC+ (Figure 1b) or MRSA (Figure 1c), respectively, corresponding to 98% or 92% killing, respectively. This moderate performance for DODAB BF contrasts with the much higher activity of the PDDA and NP dispersions against *K. pneumoniae* KPC+ or MRSA. However, against *P. aeruginosa* MDR, DODAB BF is as effective as PDDA or NPs (Figure 1a). The difference in the antimicrobial behavior of DODAB BF against *P. aeruginosa* MDR and *K. pneumoniae* KPC+, both Gram-negative microorganisms, can probably be explained by the mucus-producing feature exhibited by the *K. pneumoniae* KPC+ strain, which does not occur for the *P. aeruginosa* MDR strain tested. Regarding the lower efficiency of DODAB BF against the Gram-positive MRSA strain, it can probably be explained by the development of the resistance of Gram-positive bacteria to cationic antimicrobial agents [35]. This resistance would account for the replacement of teichoic acid by alanine, which would reduce the adhesion of cationic agents onto the bacterial cells. Another possible explanation about the differences between the antimicrobial behavior of DODAB BF against Gram-negative and Gram-positive bacteria can be related to their different cell wall structure and composition. In fact, it is known that particles bearing a cationic charge [36] or agents that increase the permeability of the outer membrane will increase the efficacy of killing of Gram-negative bacteria [37].

**Table 2:** MMC values for DODAB alone, PDDA alone or PDDA in NPs against MDR microbial strains. The single asterisk means a DODAB concentration of 2.5 μM whereas the double asterisk means a DODAB concentration of 8.0 μM.

MDR Microorganism	[DODAB] (μM)	[PDDA] (μg/mL)	[PDDA]_in NP_ (μg/mL)
*P. aeruginosa* MDR	10	1.5	2.5 *
*K. pneumoniae* KPC+	>1000	0.9	2.5 *
MRSA	>1000	5.0	8.0 **
*C. albicans* fluconazole R	>1000	0.8	2.5 *

Table 3 shows the total amount of inorganic phosphorus (Pi) in the MDR microorganisms using two different cell number densities. Considering the sensitivity of 10 nanomoles Pi for the method used (absorbance equal to 0.1) [26], it is not possible to determine the leakage of phosphorylated compounds from cells using about 10^7^ cells for bacteria or 10^5^ cells for fungi due to absorbances below 0.1 (Table 3). This emphasizes the need for using higher cell number densities for detecting the leakage of phosphorylated compounds.

**Table 3:** Determination of total inorganic phosphorus (Pi) for the MDR microorganisms at two different cell number densities.

Microorganism	Number of Cells	Absorbance	Nanomoles Pi
*P. aeruginosa* MDR	2.1 × 10^7^	0.052 ± 0.003	5.2 ± 0.3
	1.6 × 10^9^	0.441 ± 0.010	44.1 ± 1.0
*K. pneumoniae* KPC+	8.6 × 10^6^	0.045 ± 0.010	4.5 ± 1.0
	1.2 × 10^8^	0.216 ± 0.006	21.6 ± 0.6
MRSA	9.1 × 10^6^	0.032 ± 0.004	3.2 ± 0.4
	6.3 × 10^10^	0.260 ± 0.001	26.0 ± 0.1
*C. albicans* fluconazole R	8.9 × 10^4^	0.008 ± 0.002	0.8 ± 0.2
	3.3 × 10^6^	0.401 ± 0.010	40.1 ± 1.0

**Figure 3:**
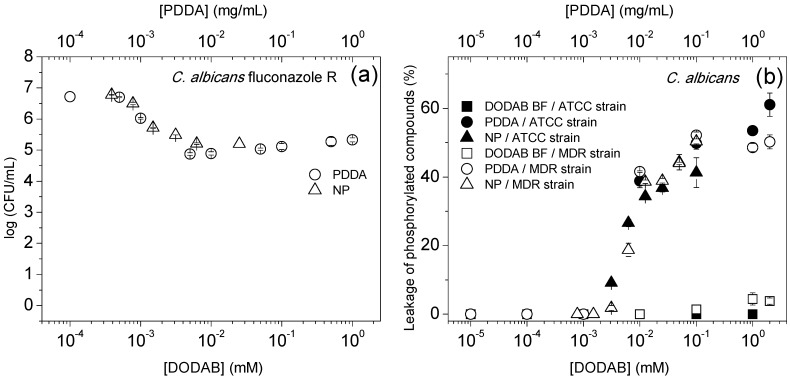
(**a**) The logarithm of CFU/mL as a function of DODAB or PDDA concentrations for the *C. albicans* fluconazole-resistant (R) strain at 5–6 × 10^6^ CFU/mL; (**b**) percentile of the leakage of phosphorylated compounds (%) as a function of DODAB or PDDA concentrations for *C. albicans* reference (ATCC) and resistant strains at 7 × 10^6^–1 × 10^7^ CFU/mL. Interactions took place for 1 h.

Therefore, absorbance readings above the detection limit of the method require at least 10^8^ bacteria or 10^6^ yeast cells to evaluate the leakage of phosphorylated compounds from the cells. Considering this, an additional experiment was performed to evaluate the antimicrobial activity of the cationic NPs and PDDA at these high cell concentrations (Figure 3). Over a range of [DODAB] or [PDDA], at cell concentrations 10–100-times higher than the ones in Figure 2, there is a two-log reduction in cell viability (Figure 3a) and detectable leakage of phosphorylated compounds (Figure 3b), suggesting an unequivocal correlation between cell death and the leakage of phosphorylated compounds. For DODAB, neither leakage (Figure 3b) nor fungicidal effect was detected over the all range of [DODAB] tested (Figure 2). This lack of DODAB BF antifungal activity agrees with previous data on the DODAB effect against an ATCC strain of *C. albicans* [17].

The maximum of cell death and the beginning of the leakage of phosphorylated compounds take place at about the same critical PDDA concentration (Figure 1, Figure 2 and Figure 3), leading to the conclusion that the cell death is directly due to cell membrane rupture with the leakage of intracellular compounds. In fact, cell lysis has often been associated with cell death by quaternary ammonium compounds [38,39], nanoparticles functionalized with chitosan [40], poly(amidoamine) dendrimers [41,42], polymers sensitive to light, as the poly(phenylene-ethynylene) (PPE)-based cationic conjugated polyelectrolytes [43], hydrogels of some self-assembled peptides containing a high arginine content [44] and other antimicrobial peptides [45,46]. The mode of action of NPs is likely to be similar to the mechanism of antimicrobial peptides for which curvature strains, localized defects and phase separation were described [45,46]. In the present case, the disintegration of the microbes is likely caused by the insertion of the linear PDDA into the cell wall and membrane causing subsequent disruption of these structures.

At the higher cell concentration used in the additional experiment (Figure 3a), the observed reduction on the antimicrobial activity of PDDA alone or in NPs was expected, since the MMC should increase with the cell number density. Furthermore, PDDA-induced cells aggregation might have happened with the protection of the inner cells in the aggregates from the biocidal effects. In fact, PDDA is commonly used as a bacteria flocculant in water treatment [47], and bacteria flocculation was previously shown to take place at high cell concentrations (starting from 10^8^ cells/mL) driven by cationic vesicles and assemblies [48].

**Figure 4:**
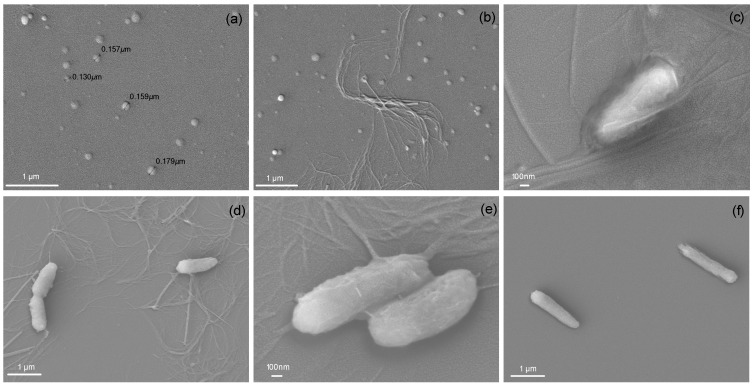
(**a**) SEM image of NPs; (**b**) SEM image of NPs and *P. aeruginosa* MDR cells at 4.4 × 10^3^ cells/mL; (**c**) SEM image of NPs and *P. aeruginosa* MDR cells at 8.9 × 10^5^ cells/mL; (**d**,**e**) SEM images of PDDA (1.0 mg/mL) and *P. aeruginosa* MDR cells at 8.9 × 10^5^ cells/mL; (**f**) *P. aeruginosa* MDR cells. Final concentrations in the NPs are 0.05 mM DODAB, 0.05 mg/mL CMC and 0.05 mg/mL PDDA.

The SEM images for NPs alone or interacting with *P. aeruginosa* MDR cells are in Figure 4a–c. For PDDA alone interacting with the bacteria, two micrographs are shown (Figure 4d,e). For bacteria only, a control is shown in Figure 4f. NPs before contact with bacterial cells show a discoidal shape and a high polydispersity (Figure 4a), in good agreement with the dynamic light-scattering data shown on Table 1 and also in agreement with our previous work [16]. However, as the cell number density increases from Figure 4b to Figure 4c, the NPs practically disappear, suggesting their disassembly upon exposure to the bacteria with unwrapping of the BF and release of the outer polymeric layers of the NPs, so that the antimicrobial PDDA could exert its outstanding microbicidal effect. Thus, the NP/cell ratio also seems to be important for visualization of the fibers on the micrographs associated with the disappearance of the NPs. At low ratios, the number of fibers on the micrographs increases, suggesting the participation of biopolymers from the cell wall in the fibers and thick bundles, which are protruding from the cells surfaces, but are able to keep a certain level of multilayer organization (Figure 4c). The importance of PDDA in the withdrawn cell wall material from the cells is well illustrated in Figure 4d,e. Fibers and bundles are also seen when PDDA interacts alone with the cells. The control showing cells alone clearly evidences the absence of fibers (Figure 4f). One should notice that at these high NP/cell or PDDA/cell ratios, the experimental conditions are completely different from those for evaluating the antimicrobial activity at low NP/cell or PDDA/cell ratios. When too many cells are seen on the micrographs, it is difficult to observe NPs, fibers and/or bundles. Therefore, the best conditions for the micrographs were obtained at high NP/cell or PDDA/cell ratios.

Although the cells’ morphology could not be assessed by SEM around the MMC, the leakage of phosphorylated compounds suggests the penetration of PDDA from NP or PDDA alone into the cell wall and cell membrane. Other authors also evidenced the penetration of cationic polymeric chains into the microbial membrane [49,50]. For antimicrobial hydrogels at a high polymer/cell ratio, the cationic polymers cause a sponge effect, suctioning out parts of the anionic microbial cell wall into the gel [51]. Besides, agents that kill by contact, disrupting or disturbing microbial cells do not appear to target the microbe metabolic activity, which is frequently associated with the development of resistance [52], and are very important for the development of non-leaching surfaces or coatings for biomedical devices [53]. For antimicrobial peptides, various resistance mechanisms are possible, including upregulation of proteolytic activity, release of extracellular polyanionic polysaccharides for electrostatically-driven scavenging and reduction of pathogen negative surface charge [54]. Although the same has not been demonstrated to any level of sophistication for cationic nanoparticles and self-assemblies, there is no reason to believe that these resistance strategies will not be activated also for other cationic antimicrobials. 

One should notice that despite the lower DODAB activity against *K. pneumoniae* KPC+ or MRSA (Figure 1b,c) or the lack of DODAB activity against yeast cells (Figure 2), the NP activity is still remarkable against these microorganisms. This points out the role of DODAB BF in the NPs as a scaffold to support the subsequent polymeric layers. Figure 4a showing the discoidal NP suggests the maintenance of the NP shape after drying and dehydration, possibly due to the two consecutive layers of CMC and PDDA stabilizing the discoidal DODAB BF in the air.

The DODAB BF/CMC/PDDA NPs have not shown hemolytic effects at doses required to kill the pathogens [17]. NPs are more interesting than PDDA alone as antimicrobials to be used *in vivo*. NPs in general are engulfed by macrophages, co-localizing with the pathogenic microbes and killing them in locus [55]. Of course, the therapeutic relevant picture is only properly achieved *in vivo*. The activity *in vivo* of the present system still requires a complete study. This will be performed in the future. For the cationic bilayer fragments alone or carrying amphotericin B or antigens, excellent therapeutic and adjuvant activity in vaccines, respectively, have already been described [56,57,58,59]. A previous study of the interaction between cationic bilayers and serum proteins revealed the colloidal stabilization achieved by similar cationic systems *in vitro* [60]. Furthermore, it is well known that nanoparticles injected in the circulation are rapidly opsonized and surrounded by the serum proteins, thereby remaining stable in the circulation until macrophages engulf them [55]. In summary, the inactivation of the present system is not expected *in vivo*.

NPs’ excellent ability to kill MDR Gram-negative microorganisms is particularly relevant due to scarce possibilities for treatment against the corresponding infections. Moreover, novel antimicrobial compounds, such as teixobactin, show excellent activity against Gram-positive pathogens, but are not as effective against Gram-negative ones [52]. At last, NPs display a broad-spectrum antimicrobial activity against Gram-positive and Gram-negative bacteria and fungi.

## 3. Experimental Section

### 3.1. Materials

Dioctadecyldimethylammonium bromide (DODAB) from Sigma Co. (St. Louis, MO, USA), carboxymethylcellulose sodium salt (CMC) with a nominal mean degree of substitution of 0.60–0.95 from Fluka (Sigma-Aldrich, Steinheim, Germany) and poly(diallyldimethylammonium chloride) (PDDA) with 20 weight % in water and 100,000–200,000 molecular weight from Sigma-Aldrich (Steinheim, Germany) were used without further purification. Difco Mueller Hinton Agar was from Becton Dickinson & Co. (Sparks, MD, USA) and Sabouraud 4% glucose agar was from Fluka Analytical (Sigma-Aldrich, Steinheim, Germany).

### 3.2. Preparation and Characterization of the Hybrid Cationic Nanoparticles

The cationic nanoparticles were built using the general layer-by-layer technique [25] specifically applied to nanostructured and cationic DODAB bilayer fragments, wrapped by one layer of the anionic CMC polyelectrolyte, which was coated by a second cationic layer of PDDA [16,22]. For this, DODAB powder was dispersed in aqueous solution (isotonic 0.264 M d-glucose solution) using an ultrasound source, which not only produces the dispersed closed bilayers, but also disrupts the closed vesicles, producing cationic open bilayer fragments (BFs) [61]. Final concentrations for each component of the nanoparticles (NPs) dispersed in the same 0.264 M d-glucose solution were 0.1 mM DODAB, 0.1 mg/mL CMC and 0.1 mg/mL PDDA.

The zeta-average diameter (Dz) or mean hydrodynamic diameter, polydispersity (P) and zeta-potential (ζ) of the assemblies were obtained by determinations using a ZetaPlus Zeta-Potential Analyzer (Brookhaven Instruments Corporation, Holtsville, NY, USA) equipped with a 570-nm laser and dynamic light scattering at 90° for particle sizing [62]. The zeta-potential was determined from the electrophoretic mobility of the assemblies (µ), the medium viscosity (η) and dielectric constant (ε) using Smoluchowski’s equation ζ = µη/ε.

### 3.3. Growth of Multidrug Resistant and Reference (ATCC) Microorganisms

Clinical isolates of MDR *P. aeruginosa*, MDR *K. pneumoniae*, producer of *K. pneumoniae* carbapenemase (KPC+), methicillin-resistant *S. aureus* (MRSA) and fluconazole-resistant (R) *C. albicans* were obtained from the Fleury Group in São Paulo, Brazil. Bacterial strains were isolated from blood samples, and the yeast strain was isolated from vaginal discharge. The *P. aeruginosa* MDR strain showed resistance to cephalosporins, carbapenems, amikacin, aztreonam and polymyxin B. The *K. pneumoniae* KPC+ strain showed resistance to penicillins, aminoglycosides, cephalosporins, carbapenems, chloramphenicol, trimethoprim-sulfamethoxazole, polymyxin B and fosfomycin. The MRSA strain showed resistance to methicillin and to all beta-lactams. Reference strains were obtained from The American Type Culture Collection (ATCC, Manassas, VA, USA): *P. aeruginosa* ATCC 27853, *K. pneumoniae* ATCC 700603, *S. aureus* ATCC 29213 and *C. albicans* ATCC 90028. Each strain was reactivated from previously frozen stocks kept at −20 °C in appropriate storage medium. The bacterial strains were plated onto Mueller Hinton agar (MHA) and incubated at 37 °C/18–24 h. The yeast cells were plated onto Sabouraud agar (SA) before incubation at 37 °C/48 h. Thereafter, some isolated colonies were transferred to an isotonic 0.264 M d-glucose solution, and the turbidity was adjusted to 0.5 McFarland [63].

### 3.4. Determination of Cell Viability

The microbicidal action of the hybrid NP against MDR bacterial and yeast strains was determined after a 1-h interaction between the assemblies and the microbial cells, in an isotonic 0.264 M d-glucose medium, over a range of DODAB and PDDA concentrations. The antimicrobial activity of DODAB BF only or PDDA only, in isotonic solutions, was also evaluated as controls. CMC was observed to be innocuous to the microbial cells. After the interaction time, the mixtures of assemblies and microorganisms were diluted up to 100,000 before plating 0.1 mL onto MHA or SA. Interactions between microbial suspensions and d-glucose were also performed as positive controls before dilution and plating. The agar plates were incubated at 37 °C/24–48 h before CFU counting. Cell viability (mean cell viability from triplicate platings ± mean standard deviation) was plotted as the logarithm of the CFU counting (CFU/mL) as a function of DODAB and PDDA concentrations. The MMC was the concentration required to achieve a reduction of the log of viable cells to zero.

### 3.5. Determination of Leakage of Phosphorylated Compounds from the Cells

The assemblies and the microbial suspensions were prepared in 1 mM NaCl solution. The lytic properties of the assemblies were evaluated from the leakage of phosphorylated compounds after a 1-h interaction between cells (*P. aeruginosa*, *K. pneumoniae*, *S. aureus* or *C. albicans* MDR and ATCC strains) and NPs, DODAB BF or PDDA. Bacterial or yeast cells were grown on MHA or SA plates, respectively, and incubated at 37 °C for 24 or 48 h, respectively. Thereafter, some isolated colonies were transferred to 1 mM NaCl solution, and the microbial suspension turbidity at 625 nm was adjusted to 0.400 for bacteria and to 1.000 for yeasts. Aliquots of microbial suspensions were transferred to 1.5-mL microtubes before they were pelleted (6000 rpm/15 min) and suspended in NPs, DODAB BF or PDDA over a range of concentrations. After 1 h of interaction, the mixtures were centrifuged (6000 rpm/15 min), and the inorganic phosphorous contents of the supernatants (Pi supernatant) were determined [26]. For determining the leakage of phosphorylated compounds, three aliquots were analyzed, and the results were shown on the curves as the mean ± the mean standard deviation. The total inorganic phosphorus content for bacterial or yeast cells was also analyzed as a positive control (Pi control). The centrifugation procedure for the cells in absence of NPs, DODAB BF or PDDA allowed the analysis of the mechanical damage caused by centrifugation to the cells and was used as a negative control. No damage to the cells was depicted from inorganic phosphorus concentrations in the supernatant close to zero. The microbial cell lysis with leakage of phosphorylated compounds was determined as % leakage = 100 × [(Pi supernatant)/(Pi control)] [27].

### 3.6. Visualization of NP, Cells and Cells/NP by Scanning Electron Microscopy

Suspensions of *P. aeruginosa* MDR and NPs (at final DODAB, CMC and PDDA concentrations of 0.05 mM, 0.05 mg/mL and 0.05 mg/mL, respectively) or PDDA (1.0 mg/mL) dispersions interacted for 1 h before centrifugation (12,000 rpm/10 min). Two different bacterial cells densities were used to interact with NPs or PDDA: 4.4 × 10^3^ and 8.9 × 10^5^ CFU/mL. The pellet was submitted to a progressive dehydration with ethanol at concentrations from 30% up to 100% [64]. After dehydration, the cells and assemblies were placed onto a silicon wafer and the ethanol evaporated at room temperature. The images were evaluated by a Jeol JSM-7401F field emission scanning electron microscope (Japan Electron Optics Laboratory, Tokyo, Japan), operated at 5 kV after sputtering the samples. Microbial suspensions in absence of the microbicidal agents were used as controls.

## 4. Conclusions

The supramolecular cationic NPs of DODAB BF/CMC/PDDA exhibit a potent broad-spectrum microbicidal effect against four clinical isolates of MDR pathogens, including Gram-negative and Gram-positive bacteria and yeast. The NPs’ mechanism of action involves the disassembly of the self-assembled NPs, leading to the appearance of fibers and/or multilayered bundles, which protrude from the cells, and to the disappearance of the NPs. These fibers visualized by SEM are thick and apparently structured as multilayers composed of the NP polymers and the biopolymers withdrawn from the cell wall. The release of PDDA from the NPs allows its insertion as a needle into the cell wall and the membrane, inducing leakage of intracellular compounds from the cells. SEM at high NP/cell ratios allows a better visualization of fibers and bundles protruding from the cells than the micrographs taken at low NP/cell ratios. The combination of the SEM and determination of leakage of intracellular phosphorylated compounds led to a more complete description of important events determining cell death. The mechanical action of suctioning the cell wall and disrupting the cell membrane makes NPs important weapons to fight infections by MDR microorganisms.
